# Penehyclidine Hydrochloride Pretreatment Ameliorates Rhabdomyolysis-Induced AKI by Activating the Nrf2/HO-1 Pathway and Allevi-ating Endoplasmic Reticulum Stress in Rats

**DOI:** 10.1371/journal.pone.0151158

**Published:** 2016-03-17

**Authors:** Wei Zhao, XuDong Huang, LiXia Zhang, XinJun Yang, LiHui Wang, YunShuang Chen, JingHua Wang, GuangLi Wu

**Affiliations:** 1 Department of Nephrology, Chinese PLA General Hospital, Beijing, the People’s Republic of China; 2 Department of Nephrology, Bethune International Peace Hospital, Shijiazhuang, Hebei Province, the People’s Republic of China; Duke University Medical Center, UNITED STATES

## Abstract

Acute kidney injury (AKI) is one of the most severe complications of rhabdomyolysis (RM). The underlying mechanisms and potential preventions need to be investigated. Penehyclidine hydrochloride (PHC) was reported to ameliorate renal ischemia-reperfusion injury, but the effect of PHC on RM-reduced AKI is unknown. In this study, we established a rat model of RM-induced AKI using an intramuscular glycerol injection in the hind limbs. Rats were pretreated with PHC before the glycerol injection, and the heme oxygenase-1 (HO-1) inhibitor ZnPP was introduced to evaluate the effect of HO-1 on RM-induced AKI. PHC pretreatment ameliorated the pathological renal injury and renal dysfunction, and decreased the renal apoptosis rate in RM-induced AKI. PHC significantly up-regulated HO-1 expression, increased HO-1 enzymatic activity and decreased the accumulation of myoglobin in renal tissues. This effect was partly inhibited by ZnPP. PHC pretreatment also effectively up-regulated nuclear factor erythroid 2-related factor 2 (Nrf2) and down-regulated glucose regulated protein 78 (GRP78) and caspase-12 at both the gene and protein levels. These results suggest that the protective effects of PHC pretreatment on RM-induced AKI occur at least in part through activating the Nrf2/HO-1 pathway and alleviating endoplasmic reticulum stress (ERS) in rat renal tissues.

## Introduction

Rhabdomyolysis (RM) is a syndrome characterized by skeletal muscle degeneration and muscle enzyme leakage [1]. The development of RM is associated with causes such as crush syndrome, exhaustive exercise, medications, infections and toxins [2–5]. Acute kidney injury (AKI) is one of the most severe complications of RM. Almost 15% of patients with RM will develop AKI [6], and 5–15% of AKI cases are attributed to RM [7].

When skeletal muscles disintegrate in RM, massive amounts of myoglobin are released into the circulation, playing a vital role in rhabdomyolysis-induced AKI. Because its molecular weight is only 17 kDa, myoglobin can easily filter through glomeruli and lead to renal tubular epithelial cell damage [8]. The mechanisms involved include oxidative stress [9], physical blockage of myoglobin casts in tubules, myoglobin metabolite toxicity and renal tubule cell apoptosis [8,10].

Nuclear factor erythroid 2-related factor 2 (Nrf2) is a transcription factor that responds to oxidative stress and regulates the expression of cytoprotective enzymes such as heme oxygenase-1 (HO-1) [11,12]. Nrf2 activation can alleviate myoglobinuric AKI [13], and HO-1 plays a cytoprotective role in a model of rhabdomyolysis-induced AKI [14]. Apoptosis, or active programmed cell death, involves two major signaling pathways: the death-receptor pathway and the mitochondrial pathway [15]. Endoplasmic reticulum stress (ERS) is another condition that can give rise to apoptosis [16]. ERS is involved in the pathogenic mechanism of myoglobin-induced renal tubule cell apoptosis [17].

Penehyclidine hydrochloride (PHC) is a selective anticholinergic agent with both antimuscarinic and antinicotinic activities. It has been widely used as an anesthetic premedication and as a treatment for smooth muscle spasm in China [18,19]. Previous studies in animals have shown that PHC reduces oxidative stress [20] and apoptosis [21]. More recently, PHC has been shown to ameliorate renal ischemia-reperfusion injury in rats [22]. In addition, studies show that an analogous anticholinergic agent called anisodamine can alleviate myocardial or renal injury by inhibiting ERS [23,24], or activating HO-1 pathway [25]. However, whether PHC can alleviate rhabdomyolysis-induced AKI and how it affects the Nrf2/HO-1 pathway and ERS in renal tissues are remain unknown.

In this study, we evaluated whether PHC could ameliorate AKI induced by rhabdomyolysis in rats and elucidated the underlying mechanisms.

## Materials and Methods

### Materials

Penehyclidine hydrochloride injection was purchased from Chengdu List Pharmaceutical Company (Lot No. 121107, Sichuan, China) and was freshly diluted to 0.05 mg/ml in 0.9% NaCl solution at the time of use. Zinc Protoporphyrin IX (ZnPP, a HO-1 inhibitor) was purchased from Sigma-Aldrich (St. Louis, MO, USA), dissolved in 0.2 M NaOH, and subsequently diluted to 2.5 mg/ml in 0.9% NaCl solution (pH 7.4) before use. Unless otherwise indicated, all other reagents used in this study were purchased from Sigma-Aldrich.

### Experimental Protocol

Ninety adult male Sprague-Dawley rats weighing 200–220 g were purchased from and maintained at the Laboratory Animals Center of the Chinese PLA General Hospital. Animals were housed with a 12-hour light dark cycle and given free access to food and water. The animal care and procedures strictly conformed to the Guide for the Care and Use of Laboratory Animals of the National Institutes of Health. This study was conducted in accordance with and was approved by the Chinese PLA General Hospital Animal Research Ethics Board. The rhabdomyolysis-induced AKI model was performed as previously described [26]. In brief, rats were lightly anesthetized with isoflurane and then injected with 10 ml/kg of 50% glycerol administered intramuscularly in equally divided dosages into each hind limb. Normal saline was administered instead of 50% glycerol for control.

Rats were randomly assigned into five groups (*n* = 18): control group; group AKI, which underwent rhabdomyolysis-induced AKI as described above; group PHC, which received 0.2 mg/kg of PHC intraperitoneally (i.p.) 30 min before glycerol injection; group ZnPP, in which 10 mg/kg ZnPP was injected i.p. 24 h before glycerol injection; and group PHC+ZnPP, in which PHC and ZnPP were separately administered as described above. All groups except the control group achieved rhabdomyolysis-induced AKI. At 1, 6 and 24 h after glycerol injection, six rats from each group were randomly selected, deeply anesthetized with pentobarbital (60 mg/kg, i.p.), and then sacrificed via exsanguination. Venous blood samples were collected, and serum was isolated by coagulation and centrifugation. The left kidney was harvested and preserved at -80°C for HO-1 enzymatic activity, real-time quantitative PCR (rt-qPCR) and Western blotting analysis, while the right kidney was harvested and fixed in 4% paraformaldehyde for pathology and immunohistochemistry examination.

### Serum Analysis

Serum urea nitrogen (BUN), creatinine (Cr) and creatine kinase (CK) were analyzed by Hitachi 7170 automated biochemistry analyzer (Tokyo, Japan). Serum myoglobin was evaluated by ELISA using a Myoglobin Rat ELISA Kit (Abcam, Cambridge, USA) as follows: samples were diluted 1:3. Then, 100 μl of standards or samples was pipetted into the wells of a microtiter plate. The plate was incubated at room temperature for 60 min. After washing the plate, 100 μl of enzyme-antibody conjugate was pipetted into each well. The plate was then incubated in the dark at room temperature for 30 min. After washing the plate, 100 μl of TMB substrate solution was pipetted into each well. The plate was subsequently incubated in the dark at room temperature for 10 min. After that, 100 μl of stop solution was immediately added to each well. The absorbance (450 nm) of the contents of each well was measured by a microplate reader (Infinite F50, TECAN, Salzburg, Austria). A standard curve was drawn, and a four parameter algorithm equation was established by Magellan software (TECAN). The myoglobin concentration in each sample was then calculated by the software.

### Assessment of Renal Pathology

Renal tissues were embedded in wax, sectioned at 4 μm and stained with hematoxylin-eosin (H&E). The tissue sections were observed by a blinded pathologist under a microscope (200× magnification, DM750, Leica, Wetzlar, Germany). Renal tubule injury was scored semi-quantitatively according to a scoring system reported previously [27]. Tubule injury was defined by necrotic lysis, tubule dilation, cast formation, sloughing of cellular debris into the tubule lumen, or naked tubule basement membrane. Tubules in the boundary area between the renal cortex and medulla and in the outer strip of the outer medulla were included. Tubular injury scores were determined by the percentage of tubules injured: 0, no injury; 1, <20%; 2, 21–50%; 3, >50%; and 4, total destruction of all epithelial cells. Five random fields for each kidney slide were examined, and the average score served as the tubular injury score of the kidney tissue sample.

### Immunohistochemistry

Renal tissues were cut into 4 μm sections, deparaffinized and permeabilized by 2 h of incubation at 65°C in 0.1 M sodium citrate. The sections were subsequently incubated for 15 min in 3% H_2_O_2_ to block the endogenous peroxidase activity. Then, the tissue section slides were incubated in a 100°C water bath for 10 min in 0.01 M PBS buffer solution. After that, the tissue sections were blocked with 2% BSA for 30 min. The sections then were incubated with anti-Nrf2 (1:200, Abcam, Cambridge, USA) or anti-myoglobin (1:250, Abcam) overnight at 4°C. We washed the slides and then incubated them with HRP secondary antibody at 37°C for 1 h. The sections were subsequently treated with DAB working solution for 4 min. Tissue sections in the boundary area between the renal cortex and medulla were observed and photographed with a microscope (500× or 200× magnification) and were semi-quantified by Image-Pro Plus 6.0 software.

### Assay for HO-1 Enzymatic Activity

The HO-1 enzymatic activity of renal tissues was analyzed as reported previously [28]. In brief, renal tissues were homogenized on ice and centrifuged at 4°C for 20 min (12,000×*g*). Supernatant (100 μl) was mixed with 0.8 mM NADPH, 0.8 mM glucose-6-phosphate, 1.0 U glucose-6-phosphate-1-dehydrogenase, 1 mM MgCl_2_, 10 ml standard rat liver tissue homogenate and 0.25 mM hemin. The mixture was incubated at 37°C in the dark for 1 h. The amount of bilirubin produced was calculated by the difference in absorption between 460 and 530 nm with a spectrophotometer. The bilirubin content per unit volume in renal tissue was represented as the HO-1 enzymatic activity of the sample.

### Real-time qPCR

For real-time qPCR analyses, total RNA was extracted from tissues in the boundary area between the renal cortex and medulla using a TRIzol total RNA extraction kit (TianGen Biotech, Beijing, China). RNA was reverse-transcribed using the PrimeScript RT reagent kit with gDNA Eraser (TaKaRa Biotechnology, Otsu Shiga, Japan). The resulting cDNA was used for PCR with SYBR Premix Ex Taq II (Tli RNaseH Plus, ROX plus) (TaKaRa Biotechnology) in triplicate on an ABI Prism 7500 Sequence Detection System (Life Technologies, CA, USA). GAPDH was used for qPCR normalization. The sequences of the primers (obtained from Invitrogen, Beijing, China) used in qPCR experiments are shown in Table 1. The quantity of mRNA was calculated from the original qPCR data according to the equation *F* = 2^−ΔΔ*ct*^. The relative mRNA expression was normalized to the fold change of the control group at 1 h after glycerol injection.

**Table 1:** Primer sequence of genes examined in this study.

Genes	Primer sequence	Product size (bp)
Nrf2	F: GGACCTAAAGCACAGCCAACACAT	177
Nrf2	R: TCGGCTTGAATGTTTGTCTTTTGTG	177
HO-1	F: CTTTTTTCACCTTCCCGAGCATC	122
HO-1	R: GGTCTTAGCCTCTTCTGTCACCCTGT	122
GRP78	F: GAATCCCTCCTGCTCCCCGT	134
GRP78	R: TTGGTCATTGGTGATGGTGATTTTG	134
caspase 12	F: CACTGCTGATACAGATGAGG	138
caspase 12	R: CCACTCTTGCCTACCTTCC	138
GAPDH	F: TGGAGTCTACTGGCGTCTT	138
GAPDH	R: TGTCATATTTCTCGTGGTTCA	138

Abbreviations: Nrf2, Nf-E2 related factor 2; HO-1, heme oxygenase 1; GRP78, glucose regulated protein 78.

### Western Blotting

Tissues in the boundary area between the renal cortex and medulla were homogenized with RIPA lysis buffer on ice. After centrifugation at 12,000 rpm and 4°C for 10 min, the protein content was determined using a BCA Protein Assay Kit (Bioword Technology, St. Louis Park, USA). The protein samples were subsequently prepared with loading buffer and heated in a water bath at 100°C for 8 min. Equal amounts (20 μg) of protein were loaded per lane, electrophoresed in a 10% SDS-PAGE gel and then transferred to PVDF membranes (Millipore, Billerica, USA). The membranes were blocked with 5% fat-free milk in TBST buffer and then incubated with primary antibodies against Nrf2 (1:500, Abcam, Cambridge, USA), HO-1 (1:1,000, Abcam), myoglobin (1:2,000, Abcam), GRP78/BiP (1:2,000, Abcam), caspase-12 (1:2,000, Abcam) and GAPDH (1:5,000, Bioword Technology, St. Louis Park, USA) at 4°C overnight. After washing, the membranes were subsequently incubated with peroxidase-labeled antibodies against mouse or rabbit IgG (1:8,000, KPL, Gaithersburg, USA) at room temperature for 1.5 h. The membrane was visualized using Biodlight ECL Chemiluminescent HRP Substrate (Bioword Technology). Moreover, to evaluate the expression of Nrf2 in nuclear protein, total protein of the boundary area between the renal cortex and medulla was separated using a nuclear and cytoplasmic protein extraction kit (Sangon Biotech, Shanghai, China), and Histone H3 (D1H2) XP Rabbit mAb (Cell Signaling Technology, Danvers, USA) served as an internal reference. Each experiment was repeated at least three times. The results were densitometrically quantified using ImageJ software.

### Assessment of Apoptosis by TUNEL

Renal tissue sections were incubated with 20 μg/ml proteinase K. After that, TUNEL staining was performed with the In Situ Cell Death Detection Kit (POD) (Roche, Mannheim, Germany) according to the manufacturer’s instructions. For cells to be considered TUNEL-positive, the nuclei were stained with DAB (brown). TUNEL-positive cells and total cells in the boundary area between the renal cortex and medulla were counted and analyzed using Image-Pro Plus 6.0 software. Five random fields per section were examined in each experiment.

### Statistical Analysis

The results are presented as the mean ± SD. Statistical analyses were performed with one-way ANOVA using SPSS 17.0. The S-N-K test was used for post hoc multiple comparisons. *P*<0.05 was accepted as statistically significant.

## Results

**Fig 1:**
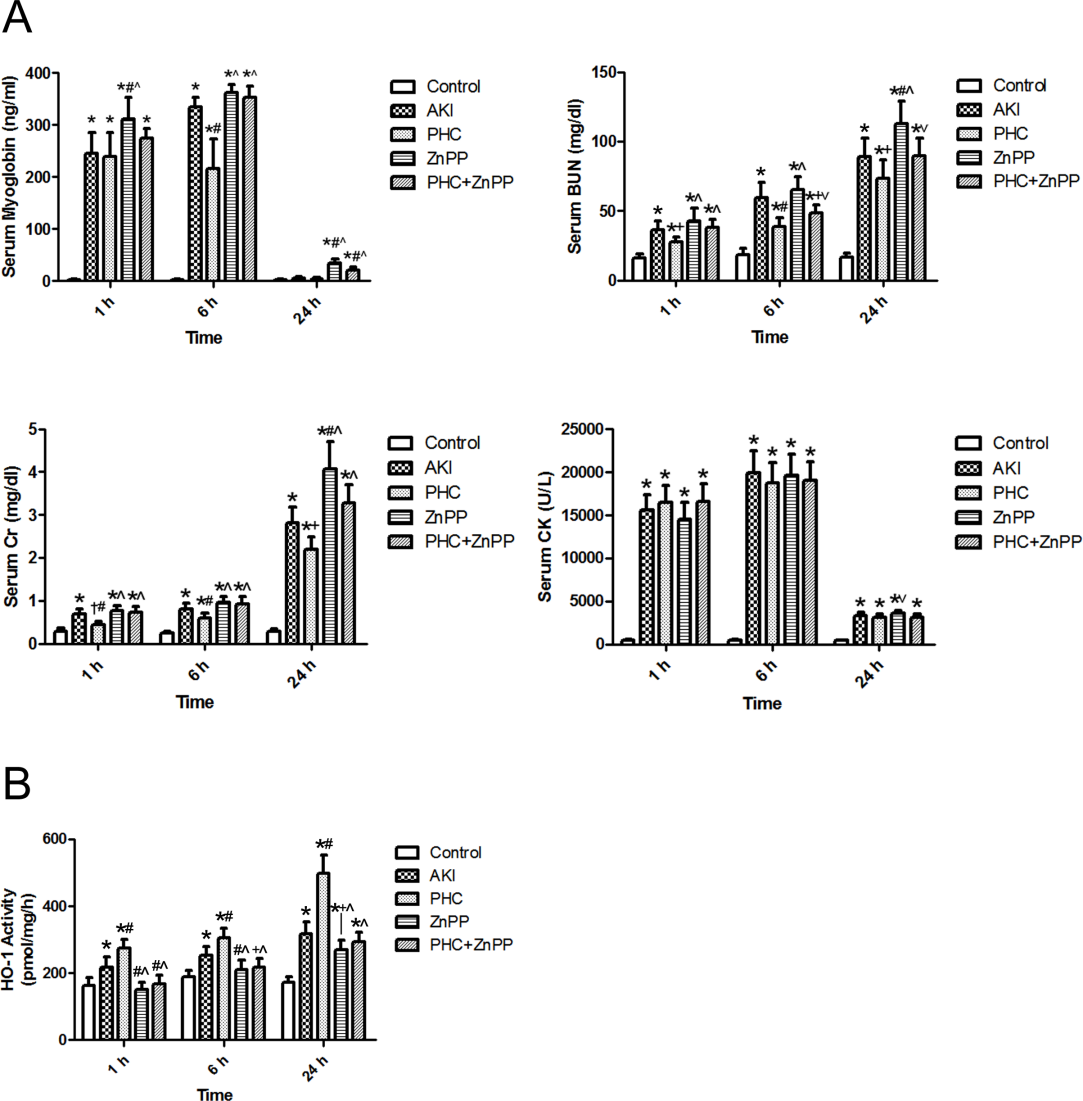
Levels of serum myoglobin, biochemical indexes and HO-1 enzymatic activity. (A) The serum myoglobin level of each group was measured by ELISA. The serum BUN, Cr and CK levels were analyzed by a biochemistry analyzer. (B) HO-1 enzymatic activity of renal tissues was expressed by bilirubin output. Each bar represents mean ± SD. Compared with simultaneous group: * *P*<0.01 or † *P*<0.05 *vs*. Control; # *P*<0.01 or + *P*<0.05 *vs*. AKI; ^ *P*<0.01 or ˅ *P*<0.05 *vs*. PHC.

Serum analysis shows, compared with group PHC, BUN and Cr were significantly higher in groups AKI, ZnPP and PHC+ZnPP at any time point (*P*<0.01 or 0.05). Levels of serum BUN and Cr were significantly higher in ZnPP group than those in groups control, AKI and PHC at 24 h (*P*<0.01). The serum myoglobin of the control group was extremely low. In group AKI, serum myoglobin levels were significantly higher than those in the control group at 1 h or 6 h (*P*<0.01). In group PHC, serum myoglobin levels were significantly higher than those in the control group at 1 h or 6 h (*P*<0.01) and were significantly lower than those in group AKI at 6 h (*P*<0.01). However, neither the difference between group AKI and group PHC at 1 h nor the differences among the control group, group AKI and group PHC at 24 h were statistically significant. The serum myoglobin levels in group ZnPP were higher than those in group AKI at 1 h and 24 h (*P*<0.01) and higher than those in group PHC at all time points (*P*<0.01). The serum myoglobin levels in group PHC+ZnPP were higher than those in group AKI at 24 h (*P*<0.01) and higher than those in group PHC at 6 h and 24 h (*P*<0.01). Compared with control group, the serum CK levels were significantly higher in other groups at any time point (*P*<0.01). The differences among CK levels in groups AKI, PHC, ZnPP and PHC+ZnPP were not statistically significant at any time point (*P*>0.05), except that the serum CK level in group ZnPP was higher than that in group PHC at 24 h (*P*<0.05) (Table 2, Fig 1A).

**Table 2:** Levels of serum myoglobin, biochemical indexes and HO-1 enzymatic activity. (mean±SD, *n* = 6).

Group	Time	Myoglobin (ng/ml)	BUN (mg/dl)	Cr (mg/dl)	CK (U/L)	HO-1 activity (pmol/mg/h)
Control	1 h	2.3±0.8	16.3±2.8	0.29±0.06	463±66	163±24
Control	6 h	2.2±0.9	18.4±4.6	0.25±0.04	514±84	188±21
Control	24 h	2.7±0.9	16.5±3.3	0.28±0.07	425±63	171±17
AKI	1 h	244.5±41.4*	36.6±6.1*	0.69±0.13*	15567±1783*	217±30*
AKI	6 h	334.3±18.4*	59.8±10.8*	0.81±0.14*	19885±2583*	253±27*
AKI	24 h	5.6±3.0	88.8±13.5*	2.80±0.38*	3308±381*	318±35*
PHC	1 h	239.6±44.7*	27.9±3.2*^+^	0.44±0.07^†^^#^	16455±1968*	274±27*^#^
PHC	6 h	215.4±56.9*^#^	38.5±6.8*^#^	0.60±0.12*^#^	18772±2368*	305±29*^#^
PHC	24 h	3.6±3.0	73.5±13.4*^+^	2.21±0.29*^+^	3140±402*	498±55*^#^
ZnPP	1 h	311.4±41.2*^#^^	42.6±9.7*^	0.77±0.12*^	14539±1984*	152±20^#^^
ZnPP	6 h	362.7±14.6*^	65.0±9.6*^	0.96±0.14*^	19645±2402*	210±30^#^^
ZnPP	24 h	34.1±8.8*^#^^	113.0±16.1*^#^^	4.07±0.62*^#^^	3569±355*^˅^	269±28*^+^^
PHC^+^ZnPP	1 h	274.7±17.3*	37.9±6.1*^	0.72±0.15*^	16561±2086*	167±26^#^^
PHC^+^ZnPP	6 h	352.6±22.4*^	48.6±5.6*^+^^˅^	0.93±0.18*^	19076±2096*	216±27^+^^
PHC^+^ZnPP	24 h	21.5±6.0*^#^^	89.6±12.6*^˅^	3.28±0.43*^	3106±374*	294±27*^

Abbreviations: BUN, urea nitrogen; Cr, creatinine; CK, creatine kinase; HO-1, heme oxygenase 1.

Compared with simultaneous group

* *P*<0.01 or

† *P*<0.05 *vs*. Control

# *P*<0.01 or

+ *P*<0.05 *vs*. AKI

^ *P*<0.01 or

˅ *P*<0.05 *vs*. PHC.

PHC pretreatment enhanced HO-1 enzymatic activity in rhabdomyolysis-induced AKI in rats. In group AKI, HO-1 activity was significantly higher than the control group at any time point (*P*<0.01). In group PHC, levels of HO-1 activity were significantly higher than those in the control group or group AKI at any time point (*P*<0.01). The levels of HO-1 activity in groups ZnPP and PHC+ZnPP were significantly lower than those in groups AKI and PHC at 1 h and 6 h (*P*<0.01 or 0.05) and that in group PHC at 24 h (*P*<0.01) (Table 2, Fig 1B).

**Fig 2:**
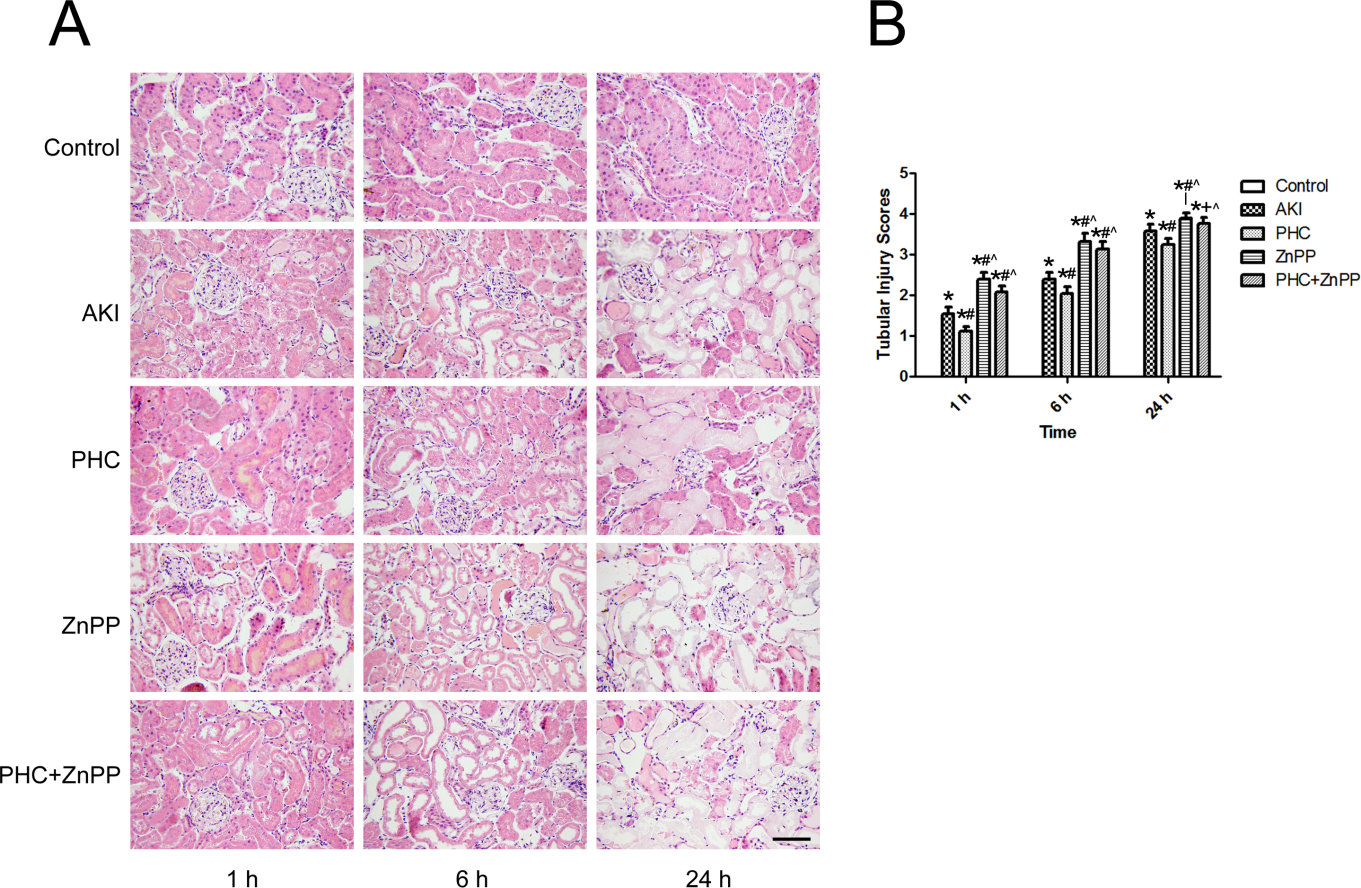
Evaluation of renal histopathology changes. (A) Renal tissue sections were stained with H&E and photographed by a microscope (200×). Control group showed no injuries. In group AKI, flocular and vacuolar degeneration of renal tubular epithelial cells was observed at 1 h. Protein casts in renal tubular lumen, tubular dilation, loss of tubular epithelial cell brush borders, and cellular debris in the tubular lumen were observed at 6 h. Necrotic lysis of tubular epithelial cells and destruction of renal tissue structure were observed at 24 h. The pathological renal changes in group PHC were more moderate than those in group AKI, while the changes in group ZnPP and group PHC+ZnPP were more severe than those in group AKI. Scale bar corresponds to 100 μm. (B) Tubular injury scores of each group. The score of the control group was zero. Each bar represents mean ± SD. Compared with simultaneous group: * *P*<0.01 *vs*. Control; # *P*<0.01 or + *P*<0.05 *vs*. AKI; ^ *P*<0.01 *vs*. PHC.

By renal H&E staining pathology examination, we found that group AKI renal tissues were remarkably damaged compared to the control group. The renal injury was aggravated over time. In group AKI, flocular and vacuolar degeneration of renal tubular epithelial cells was observed at 1 h. Protein casts in renal tubular lumen, tubular dilation, loss of tubular epithelial cell brush borders, and cellular debris in the tubular lumen were observed at 6 h. Necrotic lysis of tubular epithelial cells and destruction of renal tissue structure were observed at 24 h. The pathological renal changes in group PHC were more moderate than those in group AKI, while the changes in group ZnPP and group PHC+ZnPP were more severe than those in group AKI (Fig 2A). Because the control group had no renal tubular injuries, the tubular injury score of the control group was zero. In group AKI, the tubular injury scores were 1.54±0.16, 2.38±0.18 and 3.57±0.16 at time point 1 h, 6 h and 24 h, respectively. PHC pretreatment significantly alleviated the renal tubular injury. The scores of group PHC were 1.12±0.11, 2.04±0.16 and 3.23±0.15 at 1 h, 6 h and 24 h, respectively, which were remarkably lower than those in group AKI at any time point (*P*<0.01). The scores of groups ZnPP and PHC+ZnPP were 2.39±0.16/2.07±0.15, 3.31±0.20/3.13±0.19 and 3.89±0.13/3.76±0.15 at 1 h, 6 h and 24 h, respectively, which were significantly higher than those in groups AKI and PHC at any time point (*P*<0.01 or 0.05) (Fig 2B).

**Fig 3:**
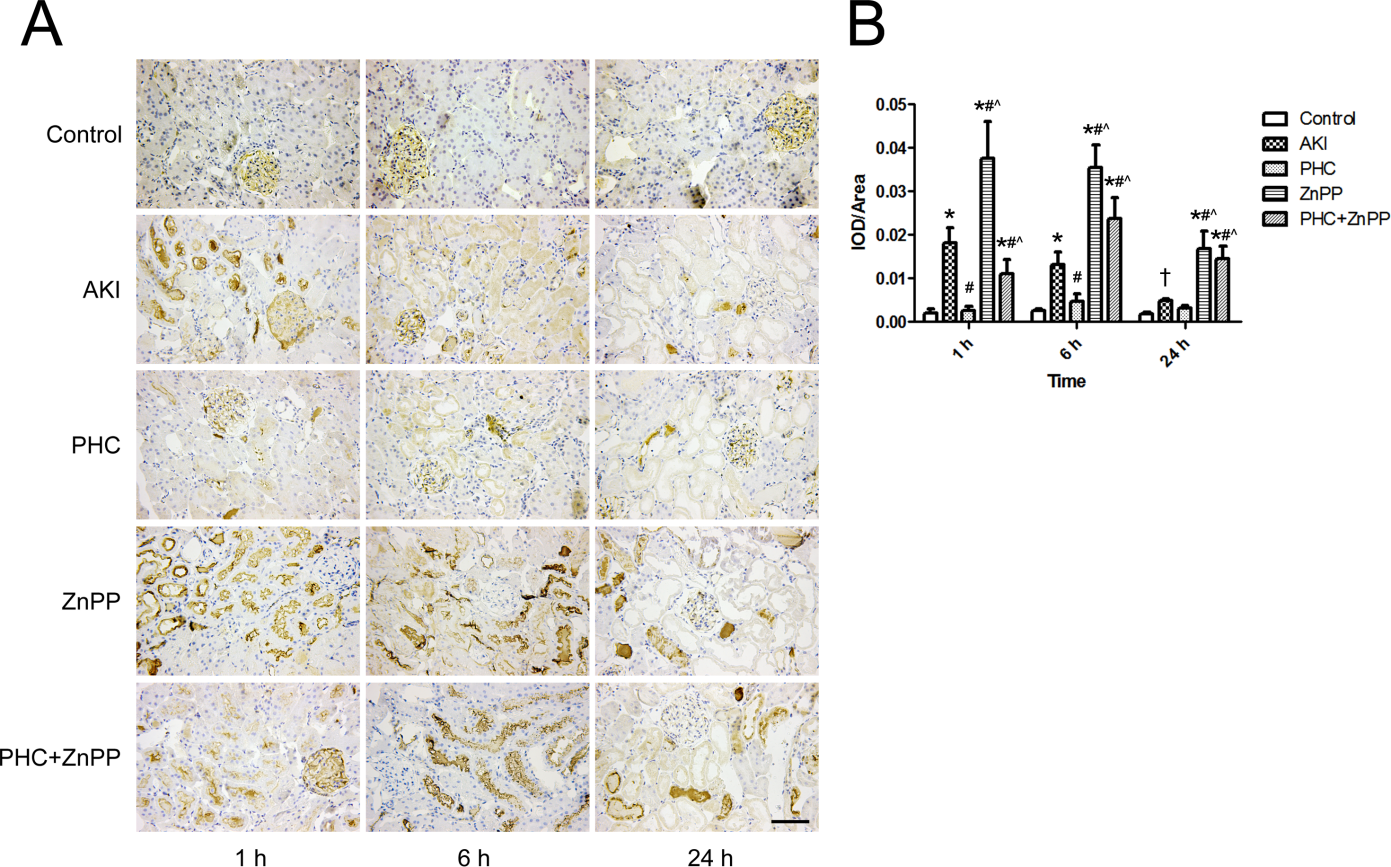
Accumulation of myoglobin in renal tissues by immunohistochemistry. (A) Renal tissue sections stained by immunohistochemistry (200× magnification). Light golden staining in the glomeruli was observed in the control group, which indicated the background expression of myoglobin in renal tissues. In groups ZnPP and PHC+ZnPP, large amounts of brown staining were observed in renal tubular lumens. In group PHC, the accumulation of myoglobin was very low. Scale bar corresponds to 100 μm. (B) Semi-quantitative evaluation of myoglobin expression represented as IOD/area. Each bar represents mean ± SD. Compared with simultaneous group: * *P*<0.01 or † *P*<0.05 *vs*. Control; # *P*<0.01 *vs*. AKI; ^ *P*<0.01 *vs*. PHC.

To determine whether PHC affected the excretion and metabolism of myoglobin in renal tissues via the HO-1 pathway, we evaluated myoglobin accumulation in renal tissues by immunohistochemistry. Light golden staining in the glomeruli was observed in the control group, indicating the background expression of myoglobin in renal tissues. In groups ZnPP and PHC+ZnPP, intense brown staining was observed in renal tubular lumens. Myoglobin accumulation was lower in group AKI than in groups ZnPP and PHC+ZnPP. In group PHC, myoglobin accumulation was very low (Fig 3A). We performed a semi-quantitative evaluation of the myoglobin expression in renal tissues using Image-Pro Plus 6.0 software. The results are represented as IOD/area. Myoglobin accumulation in group AKI was significantly higher than in the control group at all time points (*P*<0.01 or 0.05). Myoglobin accumulation in group PHC was significantly lower than in group AKI at 1 h and 6 h (*P*<0.01), although the difference at 24 h was not statistically significant. The accumulation of myoglobin in group ZnPP was significantly higher than in groups AKI and PHC at any time point (*P*<0.01). Myoglobin accumulation in group PHC+ZnPP was significantly higher than in groups AKI and PHC at 6 h and 24 h but was significantly lower than in group AKI at 1 h (*P*<0.01) (Fig 3B).

**Fig 4:**
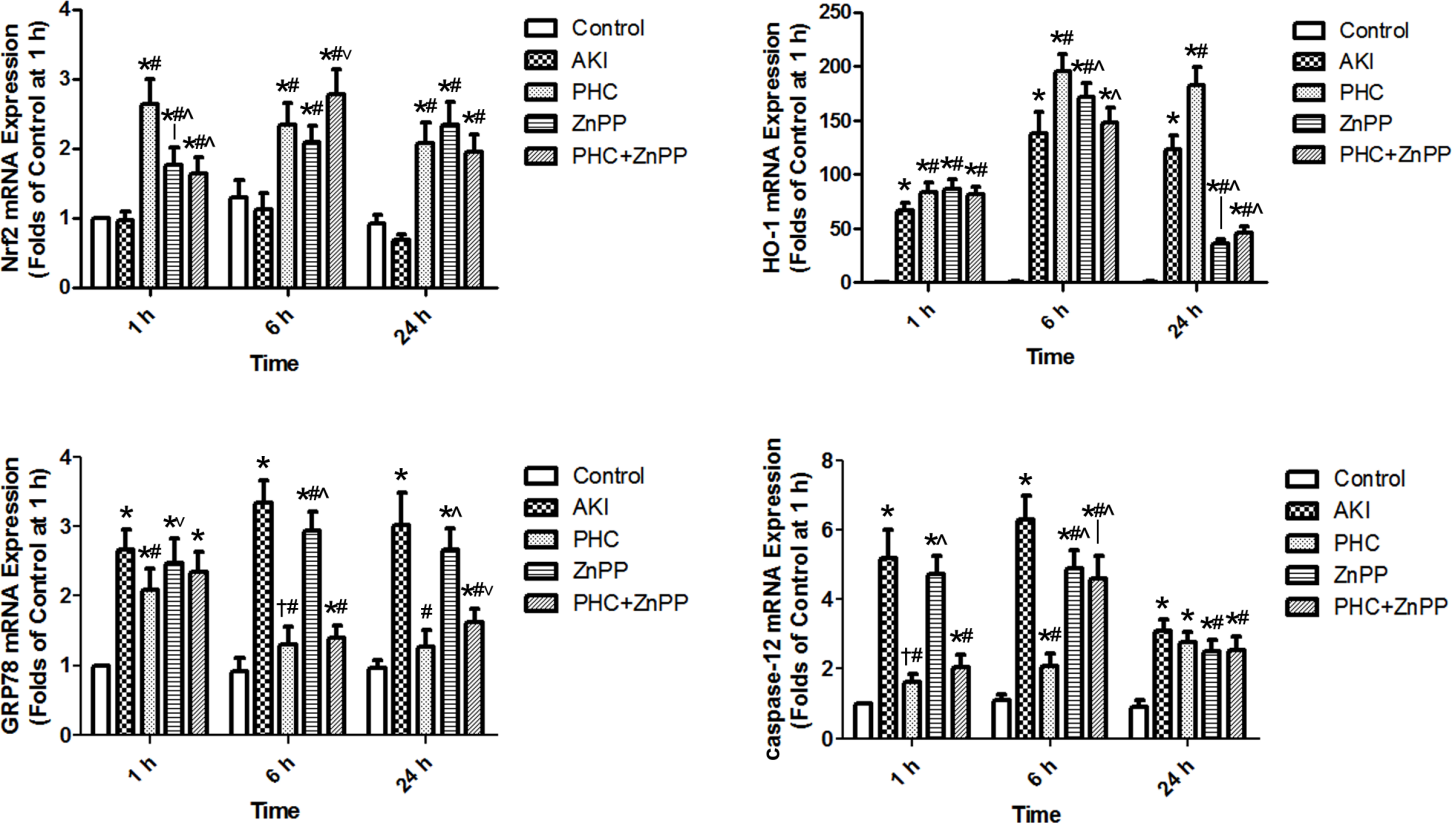
Expression of Nrf2, HO-1, GRP78 and caspase-12 mRNA in renal tissues by real-time qPCR. Each bar represents mean ± SD. Compared with simultaneous group: * *P*<0.01 or † *P*<0.05 *vs*. Control; # *P*<0.01 *vs*. AKI; ^ *P*<0.01 or ˅ *P*<0.05 *vs*. PHC.

To evaluate how PHC affected the Nrf2-HO-1 pathway and ERS genetically, we had performed real-time qPCR to determine the mRNA expression of Nrf2, HO-1, GRP78 and caspase-12. The electrophoretogram of total RNA extracted from renal tissues is illustrated as Figure A in S1 File. Nrf2 mRNA expression was significantly up-regulated in groups PHC, ZnPP and PHC+ZnPP *vs*. control group and group AKI (*P*<0.01). Nrf2 mRNA expression in groups ZnPP and PHC+ZnPP was significantly lower than in group PHC at 1 h (*P*<0.01), whereas expression in group PHC+ZnPP was higher than in group PHC at 6 h (*P*<0.05). The differences among groups PHC, ZnPP and PHC+ZnPP were not statistically significant at 24 h. Compared to the control group, HO-1 mRNA expression was significantly up-regulated in the other groups at all time points (*P*<0.01). HO-1 mRNA expression was the highest in group PHC compared to the other groups at 6 h and 24 h (*P*<0.01). GRP78 mRNA expression in group AKI was significantly up-regulated *vs*. control group (*P*<0.01) and was the highest compared to any group at 6 h (*P*<0.01). The expression of GRP78 mRNA in group PHC was lower than in group AKI at all time points (*P*<0.01) and was the lowest *vs*. groups AKI and ZnPP at all time points (*P*<0.01 or 0.05). Compared to the control group, caspase-12 mRNA expression was significantly up-regulated at all time points (*P*<0.01 or 0.05). The expression of caspase-12 mRNA in group PHC was significantly lower than in groups AKI and ZnPP at 1 h and 6 h (*P*<0.01), while there were no significant differences between group PHC and groups AKI, ZnPP and PHC+ZnPP at 24 h (Fig 4 & Figure B to F in S1 File).

**Fig 5:**
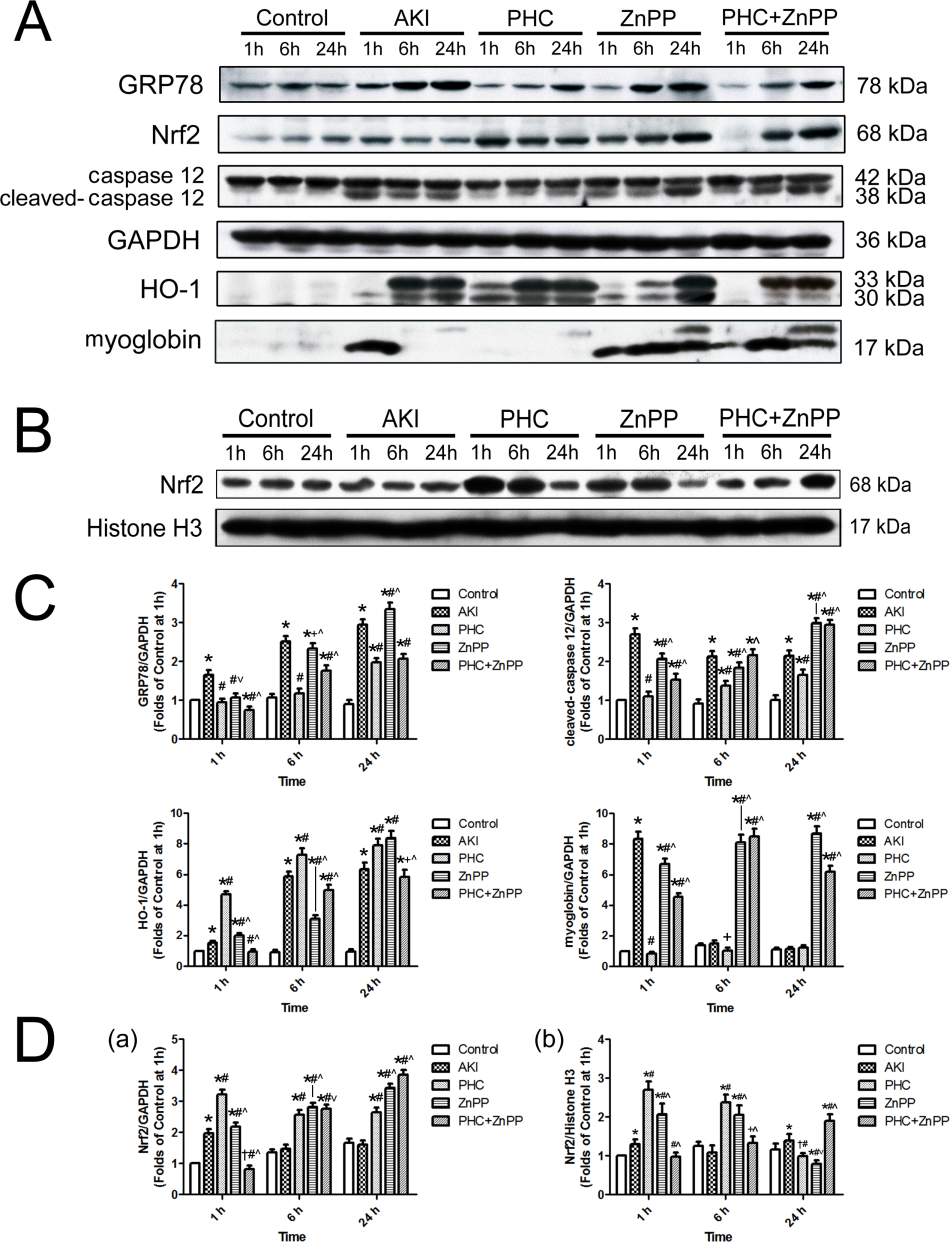
Expression of GRP78, caspase-12, HO-1, myoglobin and Nrf2 in renal tissues by Western blotting. (A) Expression in total protein of tissues in the boundary area between the renal cortex and medulla, illustrated by original immunoblotting bands and their corresponding molecular weights. The housekeeping protein GAPDH served as reference. (B) Expression of Nrf2 in nuclear protein of tissues in the boundary area between the renal cortex and medulla. Histone H3 served as reference. Each experiment was repeated at least three times. (C) Densitometrical quantitative assessment of the expression of GRP78, caspase-12, HO-1 and myoglobin in total protein. (D) Densitometrical quantitative assessment of the expression of Nrf2 a) in total protein, b) in nuclear protein. Each bar represents mean ± SD. Compared with simultaneous group: * *P*<0.01 or † *P*<0.05 *vs*. Control; # *P*<0.01 or + *P*<0.05 *vs*. AKI; ^ *P*<0.01 or ˅ *P*<0.05 *vs*. PHC.

We evaluated the expression of Nrf2, HO-1, GRP78, caspase-12 and myoglobin in total protein and Nrf2 in nuclear protein of tissues in the boundary area between the renal cortex and medulla using Western blotting. The original immunoblotting bands and their corresponding molecular weights are shown in Fig 5A and 5B. We performed a quantitative assessment to compare their differences among groups. We found that PHC pretreatment remarkably increased Nrf2 expression *vs*. group AKI in total protein at all time points and in nuclear protein at 1 h and 6 h (*P*<0.01). While the Nrf2 expression was significantly lower in group PHC than those in groups control, AKI and PHC+ZnPP in nuclear protein at 24 h (*P*<0.01 or 0.05). The expression of Nrf2 in total protein in groups ZnPP and PHC+ZnPP was significantly lower at 1 h (*P*<0.01) and was significantly higher at 6 h and 24 h (*P*<0.01 or 0.05) *vs*. group PHC. The Nrf2 expression in nuclear protein in group ZnPP was significantly higher at 1 h and 6 h and lower at 24 h than those in groups control and AKI (*P*<0.01 or 0.05) (Fig 5D). PHC pretreatment significantly up-regulated HO-1 expression *vs*. other groups at all time points (*P*<0.01), except group ZnPP at 24 h. HO-1 expression in group ZnPP was significantly higher at 1 h and 24 h and was significantly lower at 6 h than in group AKI (*P*<0.01). HO-1 expression in group PHC+ZnPP was significantly lower *vs*. group AKI at all time points (*P*<0.01 or 0.05). PHC pretreatment significantly reduced the accumulation of myoglobin in renal tissues *vs*. group AKI at 1 h and 6 h (*P*<0.01 or 0.05). The amount of myoglobin in group AKI was the highest at 1 h (*P*<0.01). In groups ZnPP and PHC+ZnPP, myoglobin amounts were significantly higher than in other groups at 6 h and 24 h (*P*<0.01). We observed that PHC pretreatment significantly down-regulated the expression of GRP78 *vs*. groups AKI, ZnPP and PHC+ZnPP at all time points (*P*<0.01 or 0.05), except group PHC+ZnPP at 24 h. GRP78 expression in group AKI was the highest compared to other groups at 1 h and 6 h (*P*<0.01 or 0.05). PHC pretreatment remarkably down-regulated the expression of cleaved caspase-12 *vs*. groups AKI, ZnPP and PHC+ZnPP at all time points (*P*<0.01). The expression of cleaved caspase-12 in groups ZnPP and PHC+ZnPP was significantly higher compared to other groups at 24 h (*P*<0.01) (Fig 5C).

**Fig 6:**
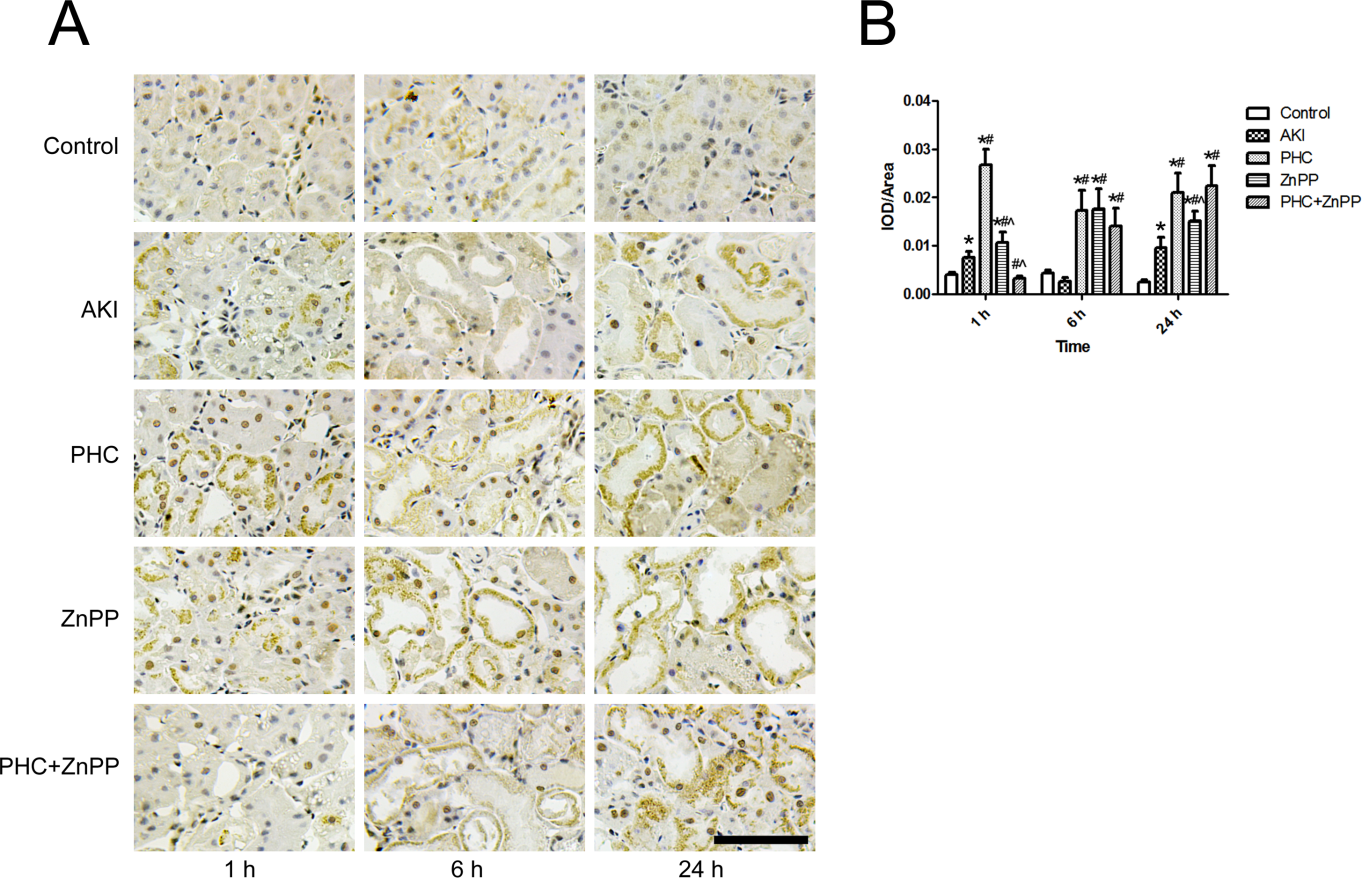
Expression of Nrf2 in renal tissues by immunohistochemistry. (A) Renal tissue sections stained by immunohistochemistry (500× magnification). Little brown yellow staining in cytoplasm of renal tubular epithelial cells was observed in the control group at all time points. Many positive nuclear staining was observed in group PHC at 1 h and 6 h. Some positive nuclear staining was observed in group AKI at 1 h and 24 h, in group ZnPP at 1 h and 6 h and in group PHC+ZnPP at 6 h and 24 h. Scale bar corresponds to 100 μm. (B) Semi-quantitative evaluation of Nrf2 expression represented as IOD/area. Each bar represents mean ± SD. Compared with simultaneous group: * *P*<0.01 *vs*. Control; # *P*<0.01 *vs*. AKI; ^ *P*<0.01 *vs*. PHC.

To illustrate the position of Nrf2 expression in renal tubular epithelial cells, we examined the renal sections by immunohistochemistry. Little brown yellow staining in cytoplasm of renal tubular epithelial cells was observed in the control group at all time points. In group AKI, some brown yellow staining in both cytoplasm and nucleus of renal tubular epithelial cells was observed at 1 h and 24 h. In group PHC, many positive nuclear staining in renal tubular epithelial cells was observed at 1 h and 6 h, and some brown yellow staining in cytoplasm was observed at 1 h and 24 h. In group ZnPP, some positive nuclear staining in renal tubular epithelial cells was observed at 1 h and 6 h, and little brown yellow staining in cytoplasm was observed at all time points. In group PHC+ZnPP, some positive nuclear staining in renal tubular epithelial cells was observed at 6 h and 24 h, and some brown yellow staining in cytoplasm was observed at 24 h (Fig 6A). The semi-quantitative evaluation of the Nrf2 expression in renal tissues represented the total expression of nucleus and cytoplasm. The expression of Nrf2 was significantly higher in group AKI than that in group control at 1 h and 24 h (*P*<0.01). In group PHC, the Nrf2 expression was significantly higher than those in groups control and AKI at all time points (*P*<0.01). In group ZnPP, the Nrf2 expression was significantly higher than those in groups control and AKI at all time points (*P*<0.01) and was significantly lower than that in group PHC at 1 h and 24 h (*P*<0.01). In group PHC+ZnPP, the Nrf2 expression was significantly lower than those in groups AKI and PHC at 1 h (*P*<0.01) and was significantly higher than those in groups control and AKI at 6 h and 24 h (*P*<0.01) (Fig 6B).

**Fig 7:**
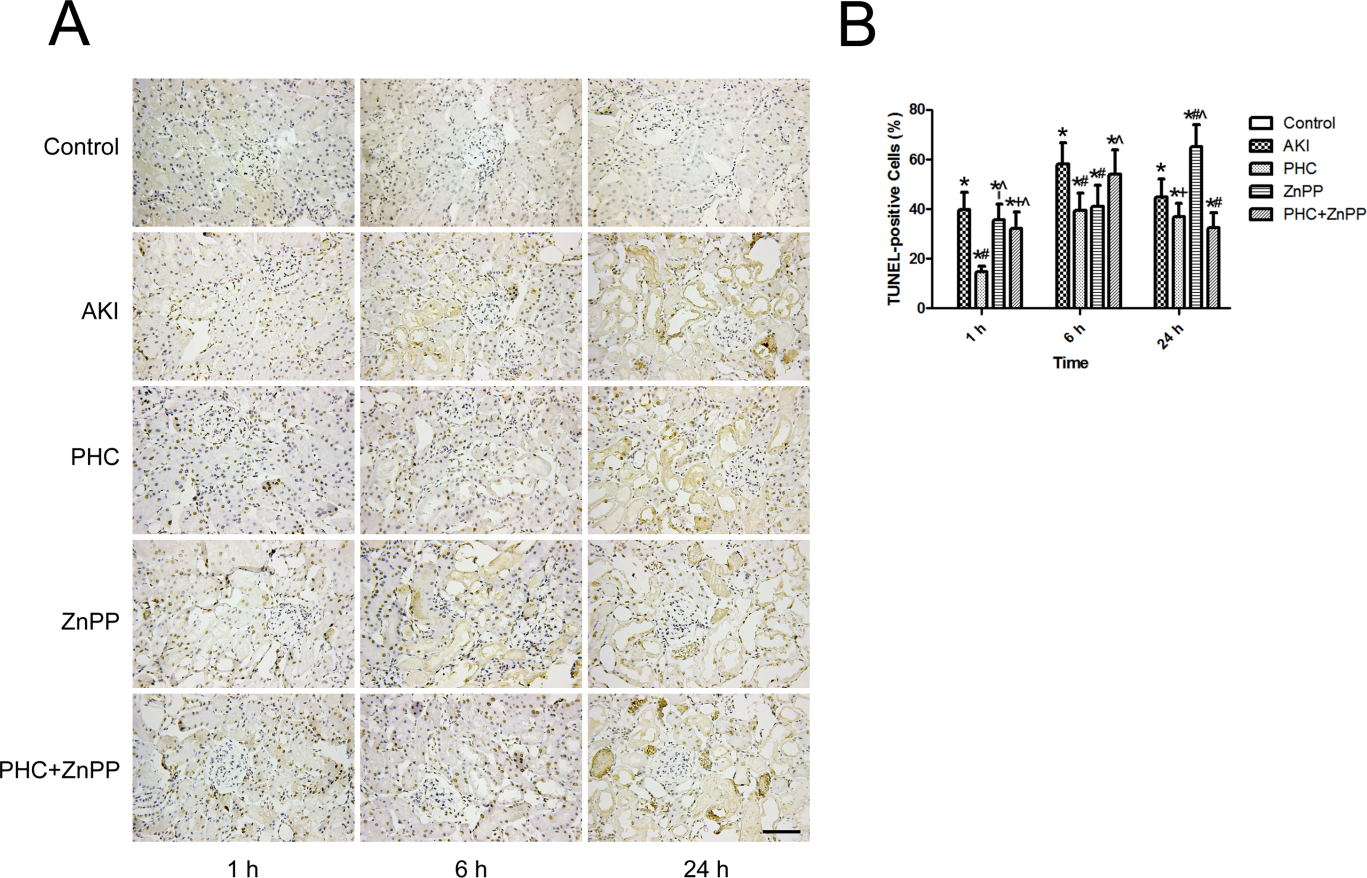
Assessment of renal tubular epithelial cell apoptosis using TUNEL assay. (A) Renal tissue sections underwent TUNEL staining (200× magnification). The nuclei of TUNEL-positive cells were stained brown, indicating apoptotic cells. Scale bar corresponds to 100 μm. (B) The levels of apoptosis were indicated as the percentage of TUNEL-positive cells among total cells. Five random fields per section were examined in each experiment. The apoptosis rate in the control group was zero. Each bar represents mean ± SD. Compared with simultaneous group: * *P*<0.01 *vs*. Control; # *P*<0.01 or + *P*<0.05 *vs*. AKI; ^ *P*<0.01 *vs*. PHC.

We evaluated the level of renal tubular epithelial cell apoptosis using the TUNEL assay. In renal sections, the nuclei of TUNEL-positive cells were stained brown, indicating apoptotic cells (Fig 7A). The levels of apoptosis were indicated as the percentage of TUNEL-positive cells among total cells. No TUNEL-positive cells were observed in the control group. Therefore, the apoptosis rate in the control group was zero. In group AKI, the apoptosis rate was 39.7±6.9%, 58.1±8.6% and 44.73±7.27% at 1 h, 6 h and 24 h, respectively. In group PHC, the apoptosis rate was 14.8±2.2%, 39.5±7.0% and 36.8±5.4% at 1 h, 6 h and 24 h, respectively, which was significantly lower *vs*. group AKI at all time points (*P*<0.01 or *P*<0.05). The apoptosis rates in groups ZnPP and PHC+ZnPP were 35.7±6.3%/32.1±6.6%, 41.2±8.4%/53.9±9.8% and 65.1±8.7%/32.6±6.0% at 1 h, 6 h and 24 h, respectively. The apoptosis rate in group ZnPP was significantly lower at 6 h and higher at 24 h than that in group AKI (*P*<0.01) and was significantly higher than that in group PHC at 1 h and 24 h (*P*<0.01). In group PHC+ZnPP, the apoptosis rate was significantly lower than that in group AKI at 1 h and 24 h (*P*<0.01 or *P*<0.05) and was significantly higher than that in group PHC at 1 h and 6 h (*P*<0.01) (Fig 7B).

## Discussion

In this study, we have shown that PHC preconditioning remarkably ameliorated rhabdomyolysis-induced AKI in rats by up-regulating the Nrf2/HO-1 pathway and inhibiting ERS in renal tissues. Conversely, inhibition of HO-1 with its specific inhibitor ZnPP caused more severe kidney damage and partly weakened the effect of PHC.

Intramuscular injection of glycerol in rats results rhabdomyolysis-induced AKI. The skeletal muscle cells lysed in the hypertonic condition, and a large amount of myoglobin was released into the circulation. Results of serum myoglobin and CK show that the muscle cells had been persistently damaged for at least 6 hours after glycerol injection. After accumulating in renal tissues, myoglobin cleaved into heme proteins and was metabolized, generating free radicals, scavenged nitric oxide and activated endothelin receptors, which synergistically induced oxidative stress, inflammation and renal tubular epithelial cell necrosis [4,6]. Because no significant difference of the level of serum CK, which is the sensitive degradation product of muscle cells, was found among groups AKI, PHC, ZnPP and PHC+ZnPP, so it can be supposed that the muscle damage among these groups has no significant difference. Myoglobin is small molecular substances like BUN and Cr, and be easily filtered through the glomerular filtration barrier. The difference of the serum myoglobin level among those groups is partly due to the different renal function of each group.

By histopathological examination, we found that renal tubular epithelial cells in renal cortex often showed focal damage when the renal pathological injury was mild or moderate, whereas the tubular epithelial cells in the boundary area between the renal cortex and medulla showed homogeneous injury whatever the renal pathological injury be. Because of the advantage of comparability, we chose the renal tissues in the boundary area between the renal cortex and medulla to perform pathological examination and protein or genetic evaluation.

Nrf2 is a master transcriptional regulator of the basal and inducible expression of a battery of defensive genes encoding detoxifying enzymes and antioxidant proteins, including HO-1 [29]. HO-1 converts heme to biliverdin via a reaction that produces carbon monoxide and liberates iron. The bile pigments and carbon monoxide can play a cytoprotective role through their antioxidant effects [30–33]. Because of the effect on heme protein, we hypothesize that HO-1 is of vital importance in the catabolism of accumulated myoglobin in renal tissues. In this study, we found that HO-1 was significantly up-regulated at both gene and protein levels in rhabdomyolysis-induced AKI at all time points, indicating that HO-1 was a rapid response factor to myoglobin in renal tissues *in vivo*. That is consistent with previous studies [14]. With intervention by PHC or ZnPP, HO-1 enzymatic activity was associated inversely with the accumulation of myoglobin in renal tissues, but the analogous phenomenon was not observed with serum myoglobin. Furthermore, PHC pretreatment significantly up-regulated Nrf2 and HO-1 at both gene and protein levels, which was negatively correlated with the accumulation of myoglobin in renal tissues and the severity of renal injury. By Western blotting analysis in nuclear protein and immunohistochemical evaluation, much nuclear expression of Nrf2 was observed in groups PHC, ZnPP and PHC+ZnPP, which further illustrated that PHC pretreatment activated Nrf2 in renal tubular epithelial cell in rhabdomyolysis-induced AKI. PHC activation of HO-1 enzymatic activity was inhibited by ZnPP. This evidence demonstrates that PHC pretreatment ameliorated rhabdomyolysis-induced AKI by activating the Nrf2/HO-1 pathway. Moreover, ZnPP intervention also remarkably up-regulated Nrf2 mRNA, activated Nrf2 in nucleus, and significantly up-regulated HO-1 at the gene and protein levels. However, HO-1 activity was not notably increased. This suggests that inhibiting the function of HO-1 can stimulate a negative feedback loop that subsequently activates the Nrf2/HO-1 pathway. The mechanism was not identified in this study.

Recently, many studies have focused on ERS-induced apoptosis. The endoplasmic reticulum (ER) is site of synthesis and folding of secreted, membrane-bound, and organelle-targeted proteins [34]. Some pathological conditions interfere with ER function and lead to the accumulation and aggregation of unfolded proteins. This can be detected by ER transmembrane receptors such as PERK, ATF6 and IRE1, which combine with the endoplasmic reticulum chaperone glucose regulated protein 78 (GRP78). Dissociating from GRP78, the three receptors initiate the unfolded protein response (UPR) to restore normal ER function [35]. If the stress is prolonged, ERS-induced apoptotic cell death ensues.

Caspase-12 is a key mediator of the commitment phase of ERS-induced apoptosis [36]. When activated by the ERS downstream receptors IRE1, ATF-6 and calpain, activated caspase-12 cleaves caspase-3 and then executes apoptosis [37–40]. Caspase-12 is only expressed in rodents. Its human homologue is caspase-4 [41,42]. A previous study showed that caspase-12 was a specific mediator of ERS-induced apoptosis in rodents [43].

In this study, we found that GRP78 and caspase-12 were up-regulated at both the gene and protein levels in group AKI, indicating that ERS was involved in renal cell apoptosis in rhabdomyolysis-induced AKI. This was consistent with a previous study [44]. Furthermore, PHC preconditioning significantly decreased the rate of renal cell apoptosis. This was associated with the down-regulation of GRP78 and caspase-12 at the gene and protein level by PHC pretreatment. PHC can therefore ameliorate renal cell apoptosis by inhibiting the ERS pathway.

However, we did not uncover the potential relationship between the Nrf2/HO-1 pathway and ERS in this study. A previous study suggested that ERS could stimulate HO-1 gene expression and regulate cell survival [45]. This theory may partly explain the results in our study. The underlying mechanism remains to be discovered.

## Conclusions

In summary, we showed that PHC pretreatment ameliorated rhabdomyolysis-induced AKI by promoting the Nrf2/HO-1 pathway in renal tissues, which decreased the accumulation of myoglobin in the kidney and alleviated oxidative stress. We also found that PHC pretreatment decreased the renal cell apoptosis rate by inhibiting ERS and down-regulating GRP78 and caspase-12 expression in renal tissues in rats.

## Supporting Information

S1 File The electrophoretogram, amplification curve and melt curve of Real-time qPCR. (PDF)
