# Correction: Penehyclidine Hydrochloride Pretreatment Ameliorates Rhabdomyolysis-Induced AKI by Activating the Nrf2/HO-1 Pathway and Allevi-ating Endoplasmic Reticulum Stress in Rats

**DOI:** 10.1371/journal.pone.0154138

**Published:** 2016-04-18

**Authors:** The PLOS ONE Staff

The word “Alleviating” is misspelled in the title. The correct title is: Penehyclidine Hydrochloride Pretreatment Ameliorates Rhabdomyolysis-Induced AKI by Activating the Nrf2/HO-1 Pathway and Alleviating Endoplasmic Reticulum Stress in Rats. The correct citation is: Zhao W, Huang X, Zhang L, Yang X, Wang L, Chen Y, et al. (2016) Penehyclidine Hydrochloride Pretreatment Ameliorates Rhabdomyolysis-Induced AKI by Activating the Nrf2/HO-1 Pathway and Alleviating Endoplasmic Reticulum Stress in Rats. PLoS ONE 11(3): e0151158. doi:10.1371/journal.pone.0151158. The publisher apologizes for the error.
