# Supplementary material for: Penehyclidine Hydrochloride Pretreatment Ameliorates Rhabdomyolysis-Induced AKI by Activating the Nrf2/HO-1 Pathway and Allevi-ating Endoplasmic Reticulum Stress in Rats
Source: PLoS One. 2016 Mar 17;11(3):e0151158. doi: 10.1371/journal.pone.0151158 (PMC4795801; doi:10.1371/journal.pone.0151158)
Supplement: S1 File — (PDF) [file pone.0151158.s001.pdf]

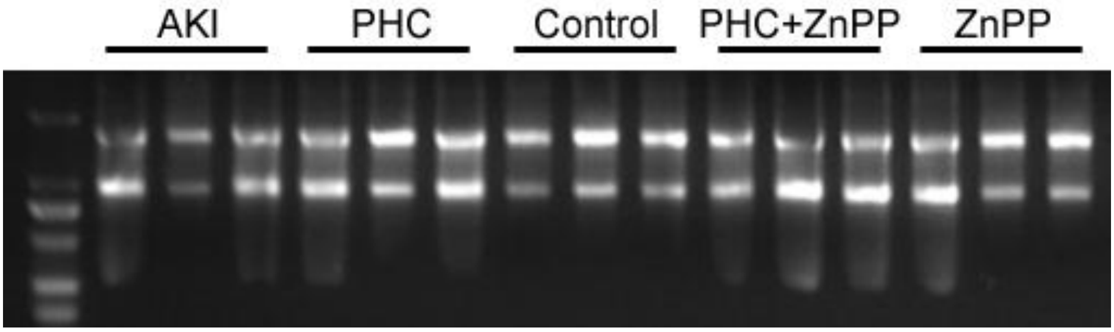

Fig. A. Electrophoretogram of total RNA extracted from renal tissues.

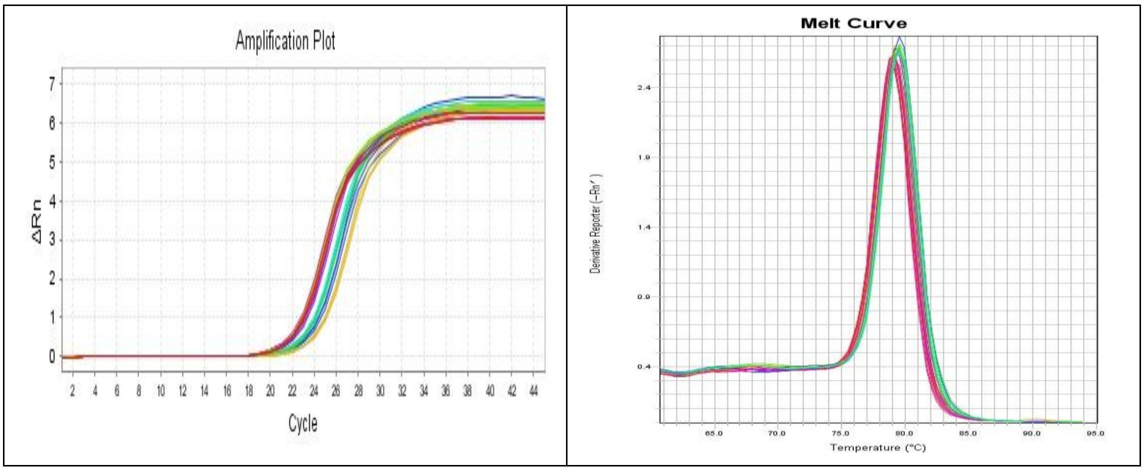

Fig. B. The amplification curve and melt curve of GRP78 mRNA.

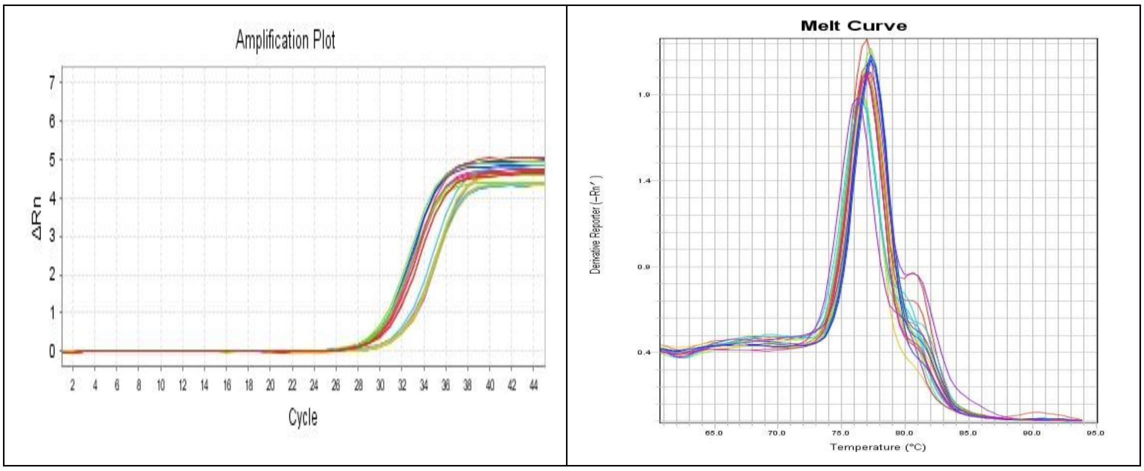

Fig. C. The amplification curve and melt curve of caspase-12 mRNA.

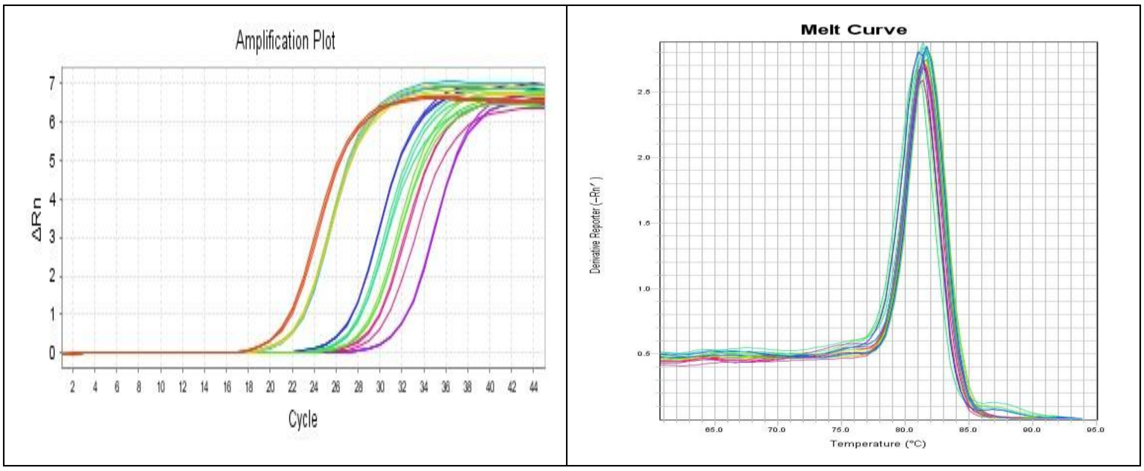

Fig. D. The amplification curve and melt curve of HO-1 mRNA.

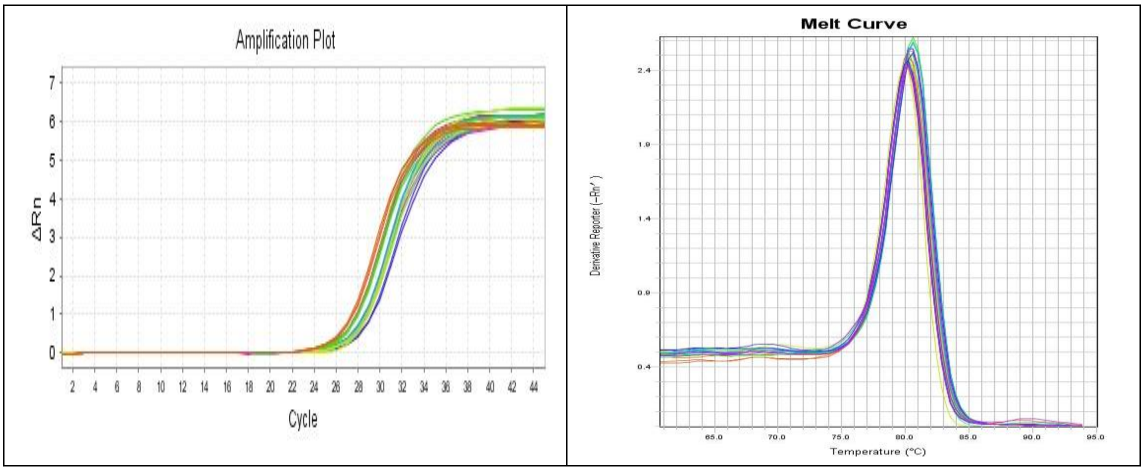

Fig. E. The amplification curve and melt curve of Nrf2 mRNA.

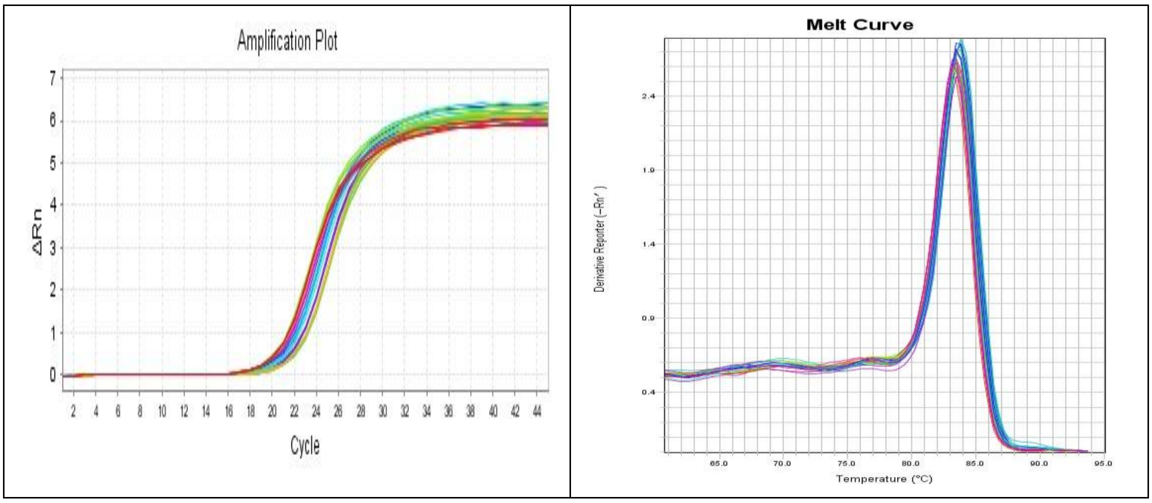

Fig. F. The amplification curve and melt curve of GAPDH mRNA.
